# Removal of Cs-137 Radionuclide by Resorcinol–Formaldehyde Ion-Exchange Resins from Solutions Simulating Real Liquid Radioactive Waste

**DOI:** 10.3390/molecules27248937

**Published:** 2022-12-15

**Authors:** Eduard Tokar, Mikhail Tutov, Svetlana Bratskaya, Andrei Egorin

**Affiliations:** Institute of Chemistry, Far Eastern Branch, Russian Academy of Sciences, Prosp. 100-letiya Vladivostok, 159, 690022 Vladivostok, Russia

**Keywords:** resorcinol–formaldehyde resin, adsorption, cesium, liquid radioactive waste

## Abstract

The efficiency of the removal of Cs-137 radionuclides with porous and non-porous resorcinol–formaldehyde resins from alkaline solutions simulating the composition of real liquid radioactive waste (LRW) streams has been evaluated. Resins were synthesized through the polycondensation of resorcinol and formaldehyde in an alkaline medium at a molar ratio of 1.8/2.2 and a temperature of 210 °C. The Cs-137 distribution coefficients on RFRs in alkaline solutions simulating LRW were above 10^3^ mL/g under static sorption conditions. In a model solution with pH 11, the full dynamic sorption capacity of non-porous RFR was 0.178 mmol/g. The values of the full dynamic sorption capacities of porous RFRs were 0.274 and 1.035 mmol/g for resins obtained with calcium carbonate and toluene as templates, respectively. When the sizes of RFR beads increased two-fold, the volume until 5% cesium breakthrough decreased by 20–40%. The most pronounced beneficial effect of the RFR’s porosity was observed at flow rates from 25 to 50 BV/h. It was shown that the negative effect of metal cations on Cs-137 uptake increases in the following order: Na^+^ < Mg^2+^ < Ca^2+^ < K^+^. The number of bed volumes of LRW-simulating solution decontaminated with RFRs until 5% cesium breakthrough was above 450; that is higher than the value of known commercially available analogs. The latter shows that the developed RFRs are promising for application in technological schemes of alkaline LRW processing.

## 1. Introduction

Liquid radioactive wastes (LRW) of various chemical and radiochemical compositions generated as a result of the operation and decommissioning of nuclear energy objects, the processing of “nuclear legacy”, and the handling nuclear emergency situations are characterized as a class of man-made radioactive waste (radwaste) to be conditioned into a solidified form prior to disposal. The most “problematic” in view of LRW management and treatment are the wastes accumulated in the course of the implementations of the atomic projects of different countries and the evaporator concentrates (EC) comprising clarified parts of heterogeneous radwaste from nuclear power plants (NPP) [1,2], whose treatment is an important ecological task. The specific features of such radwaste streams are high salinities (>100 g/L), highly alkaline mediums, and high activities with substantial contributions (up to 95%) from Cs-134/137 radionuclides with respective half-lives (T_1/2_) of 2.06 and 30.17 years [3,4]. The remaining part of the activity is provided by Co-58/60, Mn-54, Cr-51, Fe-59, Zr-95, Nb-95, I-129, I-131, Ce-144, Ru-103/106, Eu-152/154, Ba-140 and other radionuclides [5,6]. Furthermore, Cs-134/137 radionuclides’ removal from problematic LRW is additionally complicated by the presence of suspended corrosion products, complexing agents, and surfactants. That is why the problem of cesium radionuclides’ removal from LRW for subsequent disposal or radionuclides’ recycling for further applications is still rather urgent.

Selective sorption is widely used for Cs-137 removal from LRW and EC [7,8,9,10,11,12,13,14,15,16,17,18]. The main efforts were focused on the search of novel sorption materials with high chemical and radiation stabilities and improved selectivities toward Cs-134/137 radionuclides. There is a wide variety of sorption materials which can be used for the separation and concentration of cesium radionuclides under static conditions from liquid media, including LRW of different types and compositions (Table 1).

To decontaminate “problematic” LRW, sorbents based on the mixed ferrocyanides (FOC) of transition metals are the most promising among inorganic materials (Table 1) due to their high selectivities to cesium in the presence of Na^+^ and K^+^ ions at concentrations of ≥1 mol/L. Sorbents based on the FOC of transition metals (Fe, Ni, Zn, Cu, etc.) [7,8,9,10] are characterized with sufficient mechanical and radiation stabilities and are capable of removing cesium radionuclides at high rates, allowing for their industrial application in radwaste-streams’ processing. In spite of a number of advantages, transition metals FOC-based sorbents undergo peptization and dissolution in highly alkaline solutions [4,11], which limits the fields of their application and results in the increased costs of the LRW decontamination. Furthermore, in most cases, such sorbents bind cesium irreversibly and cannot be reused, so that excessive amounts of solid wastes will be accumulated.

The key role in the removal of cesium radionuclides from “problematic” LRW belongs to organic ion-exchange sorbents of the phenol type, which have been extensively applied over the recent 30 years due to their high selectivities and applicabilities in several sorption–desorption cycles. This class of ion-exchangers includes phenol-aldehyde cation-exchange resins formed via the polycondensation of phenol or its homologs with an aldehyde (formaldehyde, etc.). Moreover, phenol–formaldehyde resins (PFR) of the bifunctional type, in particular sulfo-phenol, carboxy-phenol or phosphorus-phenol, can be used for the decontamination of complex LRW from Cs-137 and Sr-90 radionuclides [19]. For example, the commercially available phenol–formaldehyde resin, Duolite CS-100, was applied for the decontamination of real low-level activity LRW from tanks at the Hanford site (USA) [12]. This cation-exchanger is characterized by its required mechanical strength and chemical stability; however, the pH of the solution has to be adjusted to 11. Furthermore, Ca^2+^ and Mg^2+^ ions interfering with radionuclides’ uptake must be preliminary removed. In addition, PFRs of the bifunctional type have low chemical stabilities in alkaline media, which resulted in radionuclide release into the decontaminated solution [19].

Researchers of the Savannah River Site (US DOE, Washington, DC, USA) patented resorcinol–formaldehyde resins (RFR) with an improved selectivity toward cesium in highly mineralized alkaline media, which is related to high content of OH^−^ groups in the ion-exchanger structure. In the repeated sorption–desorption cycles, the improved efficiency of Cs-137 uptake on RFR was accompanied by a gradual decrease of the filter capacity, which was related to the slow dissolution of the ion-exchanger due to the destruction of the polymer network in the alkaline solution during the sorption or in nitric acid during the desorption stage [13]. To decrease the hydrodynamic resistance in sorption columns, the spherically granulated RFRs (Microbeads AS) were fabricated for highly alkaline EC treatment by the deposition of the resorcinol–formaldehyde polymer on chemically stable polystyrene microspheres [14,15]. The improved accessibilities of the active sorption sites enabled one to decontaminate larger EC volumes and decrease the rates of mechanical destruction two–four-fold in comparison with “classic” RFR due to the strong binding of the resorcinol–formaldehyde polymer with polystyrene [16]. The disadvantage of such ion-exchangers consists in the four–eight-fold decrease of the efficiency of Cs-137 removal when the temperature of the solution increased from 25 °C to 45 °C, which is related to the intensification of the resin’s oxidation and the decrease of the EC flow rate down to 1.4 bed volumes per hour (BV/h).

The problem of the detrimental oxidation of ion-exchangers can be solved via the improvement of sorption kinetics in order to decrease the time of the RFR’s contact with alkaline solutions to reach a required decontamination factor under dynamic conditions. For example, the formation of a porous structure in RFR improves the accessibilities of the active exchange sites and promotes the enhancement of the sorption-selective characteristics of the cation exchanger, which is important for Cs-137’s removal from solutions with high contents of Na^+^ and K^+^ ions. The authors of [20] demonstrated the possibility of Cs-137’s removal under static conditions with porous RFR from solutions of EC simulates from NPPs with reactors of the RBMK type with the distribution coefficient 1.2 × 10^3^ mL/g.

In our earlier works, we reported the methods to fabricate porous RFR using CaCO_3_ powder [21] and toluene [22] as pore-forming templates, which were added to the liquid mixture of oligomers prior to polycondensation. The subsequent removal of templates from the polymer matrix yielded a porous structure, which was beneficial in the increase of the ion-exchange rate [21,22]. The values of the distribution coefficients of Cs-137 in LRW alkaline simulates (NaNO_3_—2.25 mol/L, NaOH—0.75 mol/L) obtained using these materials were above 10^3^ mL/g. In addition, we demonstrated earlier that the RFR stability and selectivity to Cs in alkaline solutions can be improved via the optimization of the synthesis parameters [23]. It was shown that the increase of the resorcinol/formaldehyde molar ratio in the reagent mixture from 0.6/2.2 to 1.8/2.2 and the gel solidification temperature from 130 °C to 210 °C yielded a stronger polymer network with an increased accessibility of adsorption sites. This process resulted from the condensation of thermally unstable oxygen-containing groups (–CH_2_–O–CH_2_– and –(CH_2_)-OH) and the formation of additional complex bridge structures –CH_2_-(CH_2_)_n−_. Structural modification resulting in the increase of the crosslinking degree contributed to the increase of the Cs-137 distribution coefficient. Thus, our preliminary results suggest that specific structural features and sorption characteristics of RFRs make them attractive for application in the decontamination of “problematic” LRW and EC.

**Table 1:** Comparative characterizations of sorption materials from Cs removal from liquid media.

Material	Composition	Solution Composition	pH	*S*_(*e*)_ ^1^, mg/g	K_d_ (mL/g)	Ref.
Natural aluminosilicates	Clay enriched with montmorillonite	NaNO_3_ (0.1 mol/L)	7	-	2.5 × 10^4^	[24]
Ammonium phosphomolybdate (APM)	Ammonium phosphomolybdate (APM)	NaNO_3_ (1.0 mol/L)	<7	90–140	1.3 × 10^4^–2.6 × 10^4^	[25]
APM-based composites APM	PAN/APM	HNO_3_ (1 mol/L)	<7	110	3.0 × 10^2^–6.3 × 10^4^	[26,27]
	SiO_2_/APM			80	3.1 × 10^3^	[28]
	(C_12_H_14_CaO_12_)_n_/APM			90	6.3 × 10^3^	[29]
	Zr (HPO_4_)_2_·nH_2_O/APM			10	2.5 × 10^4^	[7]
Titanates	TiO_2_-nanotubes	NaNO_3_ (0.1 mol/L)	7–9	200	1.5 × 10^3^	[30]
	TiO_2_- nanofibers/nanowire			70	10^2^	[31]
	TiO_2_-nanofilms			120	4.0 × 10^2^	[32]
	Na_2_Ti_2_SiO_7_·2H_2_O			250–590	3.1 × 10^3^	[33]
Crystalline silicotitanates	Na_2_Ti_2_SiO_7_·2H_2_O	NaNO_3_ (0.1 mol/L)	7–9	250–590	3.1 × 10^3^	[33]
TiSi-Na	(NaK)_2_ [Ti_4_(OH)O_3_(SiO_4_)_3_]·6H_2_O	EC ^2^	11.8	-	6.2 × 10^2^	[7]
		NaNO_3_ (1.0 mol/L)	6	-	6.2 × 10^4^	[7]
Transition-metal ferrocyanides	M^x^_2n_M^y^_(2−n)_[Fe(CN)_6_]	NaNO_3_ (1.0 mol/L)	6	220	2.0 × 10^5^–5.0 × 10^5^	[7]
Termoksid 35	Zirconium hydroxide/FOC Ni-K	NaNO_3_ (1.0 mol/L)	7	130	8.1 × 10^4^	[7]
ZF-N	Shabazite (clinoptilolite)/FOC Ni-K	Na^+^ (5 mol/L)	8	180	10^3^–3.0 × 10^4^	[8,9]
	Vermiculite/FOC Cu-K	NaNO_3_ (1.25 mol/L) NaOH (0.75 mol/L)	12	160	10^3^	[8,9]
Fezhel; Anfezh; Uniket	Cellulose (sawdust)/FOC Fe-K	NaNO_3_ (1.0 mol/L)	5–6	-	4.1 × 10^3^ 5.5 × 10^4^	[34]
FNS-10	Silica gel/Ni-K ferrocyanide	NaNO_3_ (1.0 mol/L)	5–6	-	7.3 × 10^4^	[34]
KU-2	Sulfo-phenol-formaldehyde cation-exchanger	NaNO_3_ (1.0 mol/L)	7	-	1–10	[35]
TOKEM	Carboxy-phenol-formaldehyde cation-exchanger	LRW ^3^	11.8	15	3.6 × 10^2^	[17]
SuperLig-644	Resorcinol-formaldehyde cation-exchanger	LRW ^3^	11.8	160	1.1 × 10^3^	[18]
Microbeads AS (Norway)	Polystyrene/Resorcinol-formaldehyde cation-exchanger	LRW ^3^	11.8	300	1.2 × 10^3^	[14,15,16]
AXIONIT RCs	Resorcinol-formaldehyde cation-exchanger	LRW ^3^	11.8	15.3	4.5 × 10^2^	[17]
		NaNO_3_ (1.0 mol/L)	11.8	-	10^3^	

^1^ Static exchange capacity (Equation (5)). ^2^ Composition—see Table 7 (LP-HLW). ^3^ Composition—see Table 6 (WWER, RBMK).

In the present work, we aim to evaluate the sorption characteristics of the earlier-developed types of RFRs with improved chemical stabilities and selectivities. We focus here on the effects of such factors as the porosities and grain sizes of the RFRs, the compositions of the solutions, and temperature on the efficiency and rate of cesium uptake. To estimate the prospects of the application of these materials for real “problematic” LRW, the efficiency of cesium removal was investigated from solutions simulating multicomponent and highly mineralized solutions.

## 2. Results and Discussion

**Figure 1:**
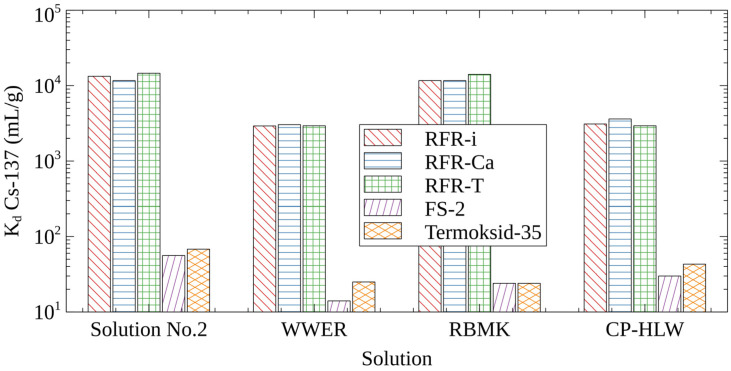
Cs-137 distribution coefficients on selective sorbents in solutions simulating different types of LRW.

In comparison with well-known FOC-sorbents, RFRs are more chemically stable in solutions with pHs > 12, which allows for their application in the removal of cesium radionuclides from alkaline liquid radioactive waste streams. Figure 1 shows diagrams demonstrating the distribution coefficient (*K_d_* Cs-137) values obtained on commercial FOC-sorbents and RFR samples in simulating solutions with different salt compositions and high pHs. It is evident that, unlike RFRs, FOC-sorbents are inefficient in alkaline solutions because of their peptization and dissolution at a high pH [4], as well as due to the decrease of their selectivity to the Cs-137 radionuclides in the presence of competing ions in amounts of >1 mol/L. The equilibrium values of *K_d_* Cs-137 for all three RFR samples demonstrate compatible values and are, on average, one–two orders of magnitude higher in comparison with FOC-sorbents.

Under static sorption conditions, the porous resins RFR-Ca and RFR-T do not demonstrate advantages in comparison with the non-porous resin RFR-i. This is explained by the same mechanism of cesium sorption on all RFRs, regardless of their porosity, and the estimation of *K_d_* Cs-137 at equilibrium, when the mass transfer was completed.

The *K_d_* Cs-137 values obtained in the model solution number two and solution simulating LWR RBMK (Table 6) are comparable. This indicates a negligible effect of K^+^ ions on the efficiency of Cs-137 removal. However, in a solution simulating borates-containing LRW WWER (Table 6), *K_d_* Cs-137 is 0.5 log unit lower in comparison with the model solution number two. Due to the fact that the negative effect of borates on the Cs-137 uptake by RFRs has not been mentioned in the literature, it deserves the separate investigation.

**Figure 2:**
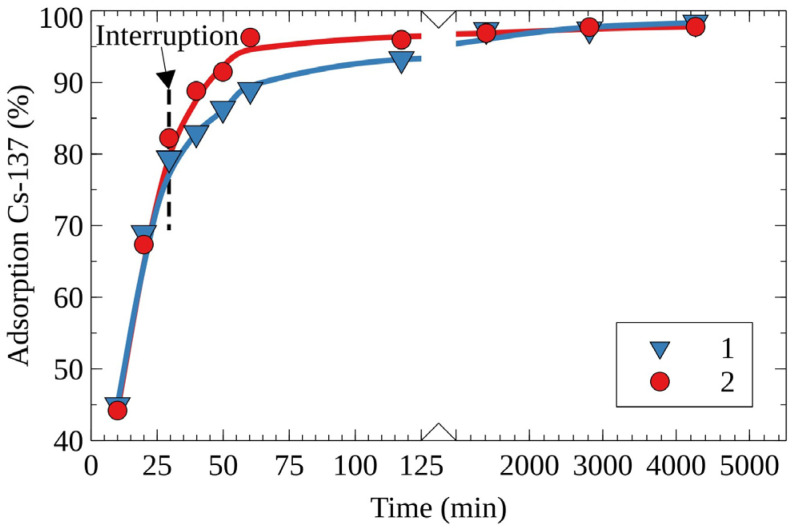
Kinetic curves of Cs-137’s removal from the solution No. 1 on the RFR-I resin, 1—the experiment without interruption (control), 2—the experiment with interruption.

To evaluate factors affecting the rate of ion exchange, the sorption experiments with an interruption were conducted. Figure 2 shows the kinetic curves of the removal of Cs-137 obtained without and with interruption of the experiment. The experiment interruption implies the removal of RFR from the model solution number one 30 min after the experiment’s start, with a subsequent return to the process after 48 h. In the case of the experiment with an interruption, the time of the attainment of the maximum efficiency of Cs-137 removal was equal to 120 min, whereas for the control experiment without interruption this value was equal to 48 h. It is evident that, within the interruption period of 48 h, Cs-137 adsorbed in the boundary layer diffused inside the ion-exchanger bead. This allows for the suggestion that the internal diffusion (gel diffusion) significantly limits the rate of cesium sorption.

**Figure 3:**
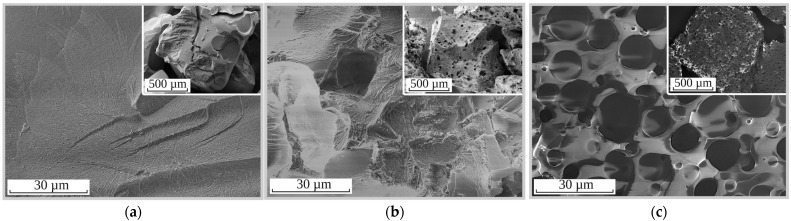
SEM images of the ion exchangers: (**a**) RFR-i, (**b**) RFR-Ca and (**c**) RFR-T.

Slow intraparticle diffusion, as the limiting stage of Cs-137 sorption kinetics, results in the decrease of the Cs-137’s removal efficiency under dynamic conditions and the increase the time of the ion-exchanger’s contact with the alkaline solutions to be decontaminated. The negative impact of poor mass transfer on RFR’s sorption performance can be eliminated through the fabrication of ion-exchange resins with porous structures. Figure 3 shows SEM images of the synthesized ion exchangers. It was demonstrated that the surface of RFR-i (Figure 3a) was smooth without cavities or channels. The porous ion-exchangers RFR-Ca (Figure 3b) and RFR-T (Figure 3c) were characterized by a spongy morphology, whose porous structures were represented by macropores.

**Figure 4:**
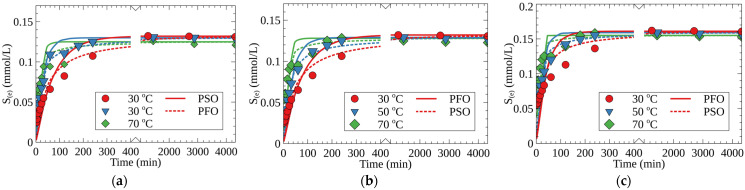
Kinetic curves of Cs-133 adsorption at different temperatures: (**a**) RFR-i, (**b**) RFR-Ca and (**c**) RFR-T; pH-11 (NaOH), V/m—500 mL/g: dots—experimental data, lines—fits with pseudo-first-order (PFO) and pseudo-second-order (PSO) kinetic models.

Figure 4 shows the kinetic curves of Cs-133 adsorption on different RFR samples under static conditions at 30, 50, and 70 °C. The calculated values of the adsorption rate constants in the pseudo-first- and pseudo-second-order kinetic models, as well as the *S*_(*e*)_ values, are given in Table 2. According to the obtained results, the kinetics of Cs-133 adsorption are best described by the pseudo-second-order model. The latter indicates that both intraparticle diffusion and chemical reaction, i.e., the ion exchange on RFR functional groups, contribute to the limiting stage of sorption kinetics. However, as compared with RFR-i, the RFR-Ca and RFR-T samples demonstrated higher adsorption rate constants of the pseudo-second order (*k*_2_), which confirms a beneficial effect of the ion-exchange resin’s porosity on the adsorption rate.

The highest *S*_(*e*)_ value was found for the RFR-T sample, and could be attributed to a more favorable configuration of the adsorption sites in the porous structure formed with toluene as a template. Although sorption rates expectedly increased with the increase of temperature, the *S*_(*e*)_ values demonstrated a gradual decrease (Table 2).

**Figure 5:**
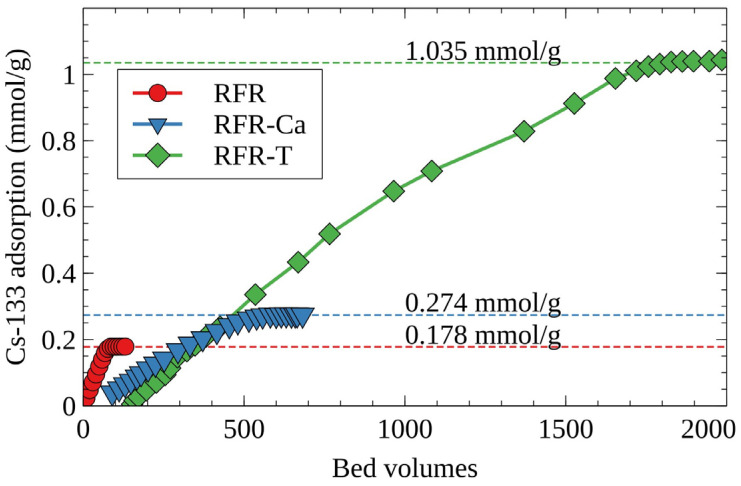
Integral curves of cesium accumulation on RFR under dynamic conditions, model solution No. 1, initial concentration of the stable isotope Cs-133—100 mg/L, grain size—0.1–0.2 mm, solution flow rate—7.5 BV/h.

Figure 5 shows the integral curves of cesium accumulation on RFR under dynamic conditions. According to the obtained results, porous ion-exchange resins surpass RFR-i on full dynamic ion-exchange capacity, which is in agreement with the *S*_(*e*)_ values obtained under static conditions (Table 2). The value of the full dynamic exchange capacity increases in the row RFR-i < RFR-Ca < RFR-T.

**Figure 6:**
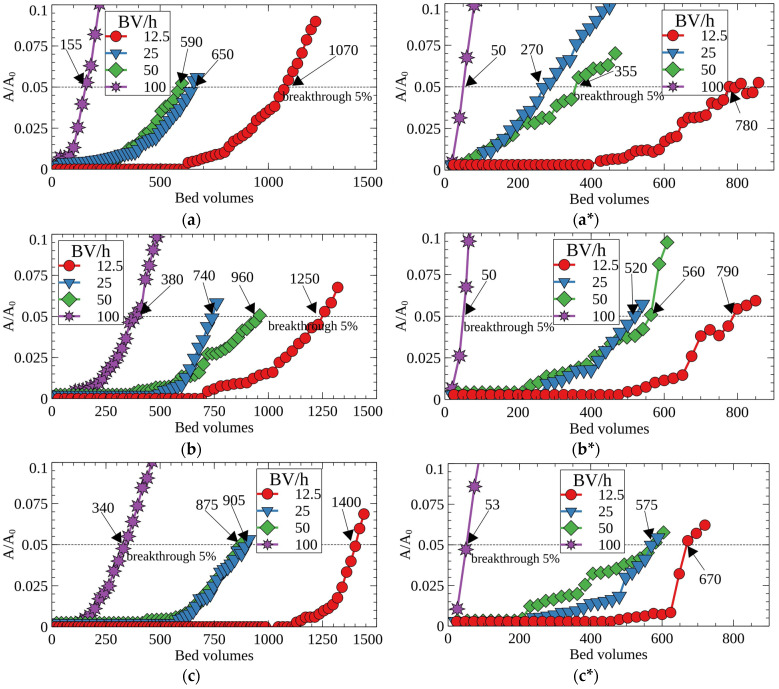
Breakthrough curves of Cs-137 sorption from the model solution No. 2 under dynamic conditions at different flow rates; (**a**) RFR, (**b**) RFR-Ca and (**c**) RFR-T, (**a**–**c**)—grain size 0.1–0.2 mm, (a*–c*)—grain size 0.2–0.5 mm.

Figure 6 shows the breakthrough curves of Cs-137 adsorption from the model solution number two on RFRs. According to the obtained results, porous ion-exchange resins demonstrated improved efficiency of the Cs-137 removal, which is evident from a significant increase of the decontaminated solution’s bed volumes until the 5% breakthrough of the radionuclide into the filtrate (effective filter cycle). A general tendency to decrease the filter-cycle value at 5% Cs-137 breakthrough was observed with the increasing flow rate. Porous resins (RFR-Ca and RFR-T) were characterized by higher values of the filter cycle due to higher ion-exchange rates in comparison with RFR-i. When the fine fraction (0.1–0.2 mm) was used, a beneficial effect of the porous structure was more pronounced. This can, most likely, be related to the increased rate of cesium mass-transfer to the functional groups in the boundary layer due to the larger surface area and the elimination of near-wall effects due to higher density of the filtering bed. The maximal positive effect from the application of porous RFRs was observed at flow rates from 25 to 50 BV/h.

**Figure 7:**
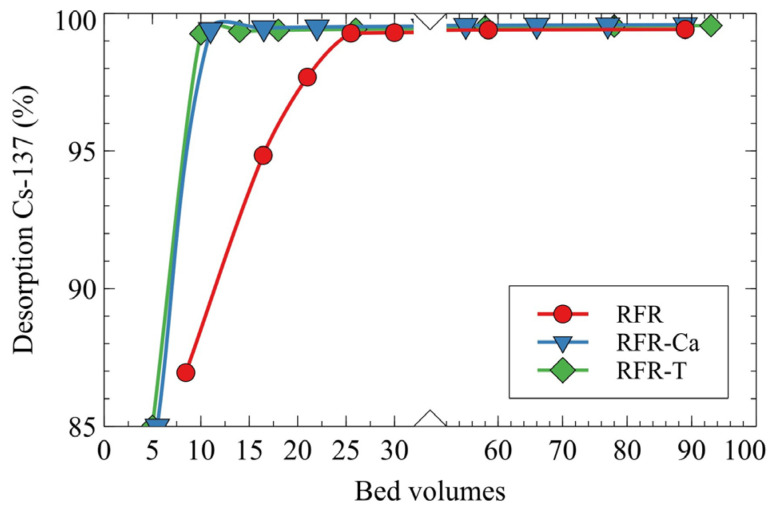
Desorption of Cs-137 by 1M HNO_3_ solution under dynamic conditions, grain size—0.1–0.2 mm, solution flow rate—7.5 BV/h.

The efficiency of Cs-137 desorption by the 1M HNO_3_ solution was evaluated under dynamic conditions (Figure 7). Porous ion-exchange resins manifested the best results in Cs-137 desorption, which were related to the better accessibility of ion-exchange groups for H^+^ ions due to the higher surface areas. Thus, the application of porous ion-exchangers is advantageous in terms of reduced volumes of secondary LRW formed during resin regeneration and reduced costs of eluent (HNO_3_).

**Figure 8:**
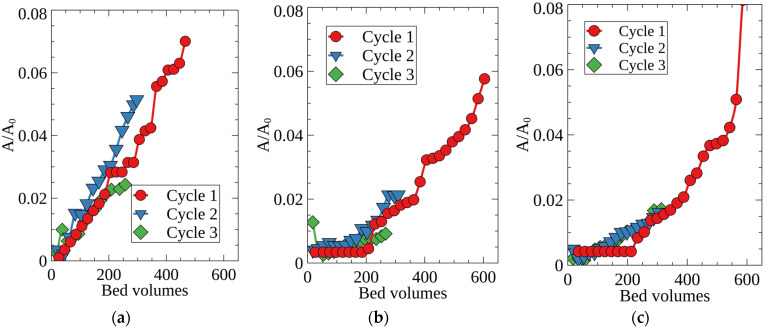
Breakthrough curves of Cs-137 sorption from the model solution No. 2. under dynamic conditions in three successive sorption cycles; (**a**) RFR, (**b**) RFR-Ca, (**c**) RFR-T, grain size 0.2–0.5 mm, solution flow rate—50 BV/h.

After regeneration with HNO_3_, RFRs can be reused to remove Cs-137 from the alkaline solutions. Figure 8 shows Cs-137 sorption in three successive cycles, in each of which at least 200 column volumes of the model solution number two were passed through the column. The experiments were carried out at flow rate of 50 BV/h, when the beneficial effect of the RFR porous structure was notable. One can see that the sorption characteristics of RFR remained unchanged at least in three successive cycles, which indicates high chemical stability of the resin. Although all types of RFRs demonstrated stable sorption characteristics in repeated sorption-desorption cycles, porous RFRs (RFR-Ca, RFR-T) are the better option for the real LWRs purification than non-porous RFRs.

**Figure 9:**
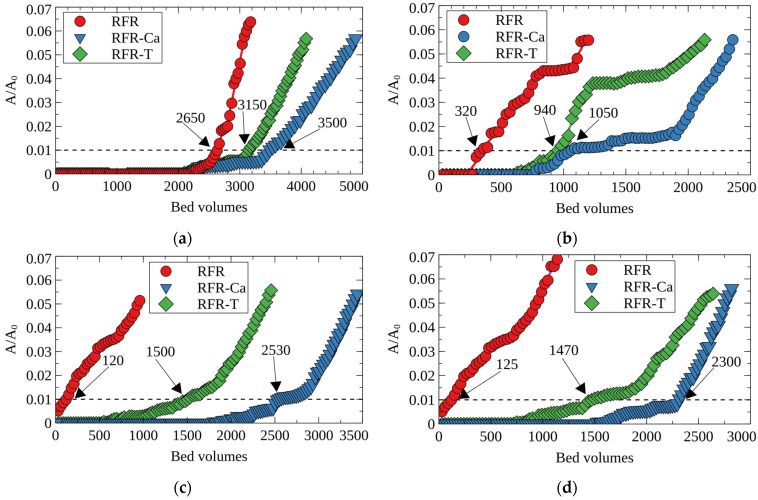
Effects of cations on the efficiency of Cs-137 removal by RFRs until the 5% breakthrough under dynamic conditions; (**a**) Na^+^, (**b**) K^+^, (**c**) Mg^2+^, (**d**) Ca^2+^, concentration 0.1 mol·eq/L, pH 9, grain size—0.1–0.2 mm, solution flow rate—12.5 BV/h.

The negative effect of alkali and alkaline-earth metal cations, which could interfere with the efficiency of Cs-137 removal from LRW, has been evaluated under dynamic conditions (Figure 9). In the experiment, the solution number three (Table 6) containing 0.1 mol·eq/L of Na^+^, K^+^, Mg^2+^, Ca^2+^ was used.

The negative effects of the Na^+^, K^+^, Mg^2+^ and Ca^2+^ cations at concentrations of 0.1 mol·eq/L on the efficiency of Cs removal increased in the order Na^+^ < Mg^2+^ < Ca^2+^ < K^+^. This order can be explained via a balance between the charge-density of the ion and the energy of its dehydration (Table 3).

From the point of electrostatic interactions between the cation and the RFR functional group [37], one can assume a higher negative effect on the cesium removal of cations with high charge densities (Mg^2+^ and Ca^2+^) because of the competitive adsorption. However, the process of the ion transfer from the solution to the ion exchanger is accompanied by a loss of the hydration shell, so that cations with low hydration energies would adsorb preferentially, which ensures the RFR selectivity to cesium [38,39]. The latter is corroborated by the fact that phenol-aldehyde cation-exchangers contain the smallest amounts of water in comparison with other groups of ion-exchange resins [40]. In view of the above facts, the K^+^ ion with the lowest hydration energy showed the strongest interference with Cs-137 uptake. Due to their high charge densities, Mg^2+^ and Ca^2+^ ions affected Cs-137 adsorption stronger than Na^+^ ions with lower hydration energies.

**Figure 10:**
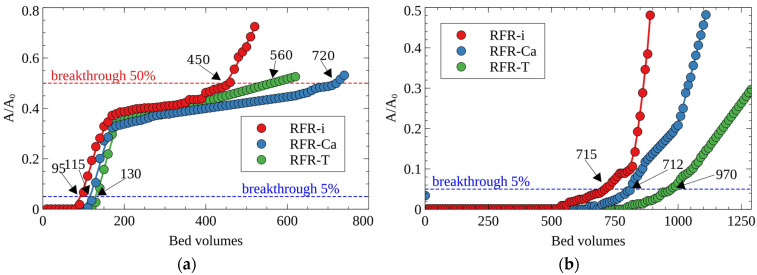
Breakthrough curves of cesium sorption from CP-HLW (see Table 7): (**a**) Cs-137 + 133 (0.05 g/L), (**b**) Cs-137, grain size—0.1–0.2 mm, solution flow rate—25 BV/h.

The presence of macro concentrations of the stable Cs-133 isotope in LRW can significantly decrease the filter cycle value, which has to be taken into account in practice. Figure 10 demonstrates the breakthrough curves of Cs-137’s uptake from CP-HLW in the presence of Cs-133 (Figure 10a) and without Cs-133 (only micro-concentrations of Cs-137) (Figure 10b). One can notice that the addition of a stable isotope Cs-133 reduces six–seven-fold the value of the efficient filter cycle until 5% Cs-137 breakthrough due to the increased rate of sorption sites’ occupation. Despite this effect, porous RFRs surpassed RFR-i, whereas RFR-Ca resin demonstrated the best performance.

One should mention that for the decontamination of the solution simulating the clarified fraction of LRW containing Cs-133/137 (CP-HLW), the developed RFRs demonstrated the values of the effective filter cycle, which surpassed by two–four-fold the values available in the literature for analogs of various trademarks and grades (Table 4).

## 3. Materials and Methods

The synthesis of non-porous RFR was carried out according to the scheme described in [23] through the interaction of an alkaline solution of resorcinol and formalin under the conditions shown in Table 5. The solidification of the ion-exchange resin was performed in air atmosphere at 210 °C for 6 h. The resulting ion-exchange resins were ground and transformed into the H^+^-form through sequential washing by HNO_3_ (0.5 mol/L)–NaOH (0.5 mol/L)–HNO_3_ (0.5 mol/L) solutions under dynamic conditions. The final ion-exchange resin marked as “RFR-i” consisted of dark-brown granules of an irregular shape.

The porous morphology of the ion-exchange resins was achieved using CaCO_3_ or toluene as pore-forming agents, as described in detail in [21,22], respectively. The synthesis conditions are shown in Table 5. The resin synthesized by the addition of a CaCO_3_ powder in 10 wt.% of the total weight of the reaction mixture was marked as “RFR-Ca”. The sample synthesized by the addition of toluene in 25 wt.% of total weight into the liquid oligomeric mixture was marked as “RFR-T”. For the sorption experiments, the ion-exchange resins were transferred into the H^+^-from.

The following commercially available sorption materials were used in this work for comparison: FS-2—ferrocyanide sorbent with a Cu-K ferrocyanide content of 45–50 wt.% (ALLIANCE GAMMA Ltd., Moscow, Russia, produced under Technical Conditions 6-09-40-573–84) [4]; Termoksid-35—spherically granulated sorbent based on nickel ferrocyanide with a content of 32–36 wt.% of zirconium hydroxide support (Termoksid Ltd., Moscow, Russia, produced under Technical Conditions 6200-305-12 342 266–98) [41].

The investigation of the efficiency of Cs-137 removal and Cs-137 distribution coefficients was carried out under static conditions as follows: a weight sample of an ion exchanger was preliminarily held for 12 h in a model solution without Cs to establish the equilibrium at a volume/weight ratio of1000 mL/g. Thereafter, the solution was replaced by a new one spiked with Cs-137 (500–100 Bq/mL) or containing a stable Cs-133 isotope of a preset concentration of 50 mg/L.

To determine the Cs-137 distribution coefficient, the time of RFR’s contact with the model solution (Table 6 and Table 7) was at least 24 h. Thereafter, the solution was separated from RFR by filtering on an ash-free blue-ribbon filter, and the residual content of the Cs-137 radionuclide was determined.

The Cs-137 distribution coefficient (*K_d_*) was calculated according to the Formula (1):

(1) $$K_{d}=\frac{\left(A_{0}-A_{1}\right)}{A_{1}}\times \frac{V}{m}$$

where *A*_0_ is the solution’s initial activity (Bq/mL), *A*_1_ is the solution’s final activity (Bq/mL), *V* is the solution’s volume (mL), and *m* is the sorbent’s weight (g).

To evaluate the effect of the mass–transfer limitation on the adsorption rate, the experiment was interrupted as follows: RFR was separated from the model solution and returned after a preset period. The efficiency of the Cs-137 removal (%) from the model solution was calculated using Formula (2):

(2) $$Ads\left(\%\right)=\left(1-\frac{A_{1}}{A_{0}}\right)\times 100$$

Table 6 and Table 7 show the characteristics of the model solutions used in the present work.

The kinetic parameters were evaluated at temperatures of 30, 50, and 70 °C under constant stirring using a thermostated shaker. Within preset periods, the solution aliquots were sampled, and the residual concentrations of the Cs-133 stable isotope in the solution were determined.

To calculate the kinetic parameters of the Cs sorption, the experimental values in *S*_(*t*)_ = *f*(*t*) coordinates (*S*_(*t*)_—the amount of the adsorbed cesium at time *t* (mmol/g)) in the time (min)) were fitted using kinetics equations of the pseudo-first (3) and pseudo-second (4) orders:

(3) $$S_{\left(t\right)}=S_{\left(e\right)}\times \left(1-exp^{-k_{1}\times t}\right)$$

(4) $$S_{\left(t\right)}=\frac{k_{2}\times S_{\left(e\right)}{}^{2}\times t}{1+k_{2}\times S_{\left(e\right)}\times t}$$

The content of the cesium adsorbed at the time *t* (*S*_(*t*)_) and the static adsorption capacity at equilibrium (*S*_(*e*)_) were calculated using Equation (5).

(5) $$S=\frac{\left(C_{0}-C_{1}\right)\times V}{m}$$

where *k*_1_, *k*_2_ are the adsorption rate constants in the kinetics models of the pseudo-first and pseudo-second orders, respectively (min^−1^ and mg/g·min), *C*_0_ is the initial concentration of cesium (mmol/mL), and *C*_1_ is the equilibrium concentration of cesium (for *S*_(*e*)_) or the residual concentration of cesium in the solution at the time (for *S*_(*t*)_) (mmol/g).

The breakthrough Cs-137 adsorption curves were built in the coordinates f(*A*/*A*_0_) = *BV*, where *BV* is the bed volume calculated as the ratio of the sorbent volume in the column (mL) to the volume of the fed solution (mL), *A*_0_ is the initial Cs-137 activity in solution (Bq/mL), and *A* is the residual Cs-137 activity in the solution (Bq/mL).

To evaluate the effects of competing ions (Na^+^, K^+^, Ca^2+^, and Mg^2+^) on the efficiency of Cs-137’s removal under dynamic conditions, the model solutions number three (Table 6) containing chloride salts of respective metals in amounts of 0.1 mol·eq/L were prepared. The model solutions were fed through a layer of the swollen RFR of a volume of 1 mL at a rate of 12.5 BV/h.

The completeness of Cs desorption in dependence on the feed rate of the eluent was evaluated under dynamic conditions as follows. The resin was preliminarily saturated with Cs-137 under dynamic conditions, after which a solution of HNO_3_ (eluent) of a concentration of 1 mol/L was fed at a rate of 7.5 BV/h, and the filtrate was collected in fractions to determine the activity on the Cs-137. The Cs-137 desorption under dynamic conditions was calculated according to Formula (6):

(6) $$Desorption\left(\%\right)=\left(\frac{\sum_{1}^{i}V\times A_{el}}{A_{f}}\right)\times 100$$

where *A_el_* is the Cs-137 activity in the eluate (Bq/mL), *V* is the eluate volume (mL), *A_f_* is the activity of the Cs-137 removed at the sorption stage (Bq), and *i* is the eluate fraction number.

The Cs-137-specific activity (photopeak energy: 662 keV) was determined by the direct radiometric method using an RKG-AT1320 gamma-radiometer equipped with a NaI(Tl) detector measuring 63 × 63 mm (RPE Atomtech, Minsk, Republic of Belarus). The ion-exchange resins’ images were obtained using a Hitachi TM-3000 scanning electron microscope (Hitachi, Ibaraki, Japan). The contents of the stable Cs-133 isotope in solutions were determined by the method of atomic absorption spectrometry using a AA-7000 spectrometer (Shimadzu, Kyoto, Japan).

The experimental data were processed using the Veusz software (ver. 3.4) [42].

## 4. Conclusions

The RFRs suggested in the present work for the decontamination of LRW with a complex composition surpass, in terms of efficiency of Cs-137 removal, sorbents based on FOC of transition metals and other known RFRs, including those commercially available. The porous materials, RFR-Ca and RFR-T, synthesized with CaCO_3_ and toluene as templates, demonstrated increased cesium-exchange rates, which were related to the high accessibilities of the ion-exchange sites and the improved mass transfer in comparison with a non-porous analog.

The optimal parameters of RFR’s application were found in the series of sorption experiments under dynamic conditions using solutions simulating different types of LRW. It was found that the porous samples RFR-T and RFR-Ca have higher values of the effective filter cycle (until 5% and 50% breakthrough) under dynamic conditions, in comparison with non-porous RFR-i synthesized at similar conditions. The efficiency of Cs-137 desorption from the porous RFRs was higher, allowing for the minimization of the secondary waste volume. It has been established that RFR-Ca was the most preferable choice of material for decontamination of highly alkaline LRW (pH ≥ 13).

The developed RFRs can be recommended for the decontamination of LRW of a complex chemical composition from Cs-137 radionuclides. Their application will enable one to improve the sorption technologies of a LRW stream’s treatment and reduce possible radioecological risks in waste management.

## Figures and Tables

**Table 2:** Parameters of the pseudo-first (PFO) and pseudo-second (PSO) orders Cs-133 sorption kinetics.

Name	Model	Parameter	Temperature, °C		
			30	50	70
RFR	PFO	*S*_(*e*)_ (mmol/g)	0.132 ± 0.02	0.130 ± 0.02	0.125 ± 0.02
		*k*_1_ (min^−1^)	0.032 ± 0.001	0.033 ± 0.001	0.061 ± 0.001
		*R* ^2^	0.986	0.988	0.969
	PSO	*k*_2_ (g(mmol·min)^−1^	0.171 ± 0.001	0.445 ± 0.001	0.918 ± 0.002
		*R* ^2^	0.984	0.995	0.989
RFR-Ca	PFO	*S*_(*e*)_ (mmol/g)	0.132 ± 0.02	0.129 ± 0.02	0.128 ± 0.02
		*k*_1_ (min^−1^)	0.013 ± 0.001	0.027 ± 0.001	0.067 ± 0.001
		*R* ^2^	0.968	0.987	0.968
	PSO	*k*_2_ (g(mmol·min)^−1^	0.162 ± 0.001	0.369 ± 0.001	1.03 ± 0.01
		*R* ^2^	0.986	0.995	0.987
RFR-T	PFO	*S*_(*e*)_ (mmol/g)	0.161 ± 0.02	0.159 ± 0.02	0.155 ± 0.02
		*k*_1_ (min^−1^)	0.024 ± 0.001	0.043 ± 0.001	0.141 ± 0.001
		*R* ^2^	0.941	0.973	0.970
	PSO	*k*_2_ (g(mmol·min)^−1^	0.272 ± 0.001	0.529 ± 0.001	1.66 ± 0.01
		*R* ^2^	0.968	0.988	0.989

**Table 3:** Effective and hydrated cation radii and dehydration e6nergy [36].

Cation	Cs^+^	K^+^	Na^+^	Mg^2+^	Ca^2+^
Ion radius, nm	0.174	0.138	0.102	0.072	0.106
*Z*^2^/r (1/nm)	5.62	6.99	9.35	61.54	36.7
Hydrated radius, nm	0.329	0.232	0.276	0.430	0.420
Dehydration energy, kJ/mol	−376	−321	−405	−1922	−1650

**Table 4:** Comparative characteristics of sorption materials for cesium removal from the clarified LRW fraction under dynamic conditions.

Sorption Materials Grade (™)	Effective Filter Cycle, BV ^1^	Ref.
AXIONIT RCs (Russia)	185	[18]
Microbeads AS (Norway)	180	[13]
SuperLig-644	225	[38]
RFR-i	450	This work
RFR-Ca	720	
RFR-T	560	

^1^ The number of decontaminated bed volumes until the cesium breakthrough into the filtrate, >50%.

**Table 5:** RFR synthesis conditions and samples notations.

	RFR-i	RFR-Ca	RFR-T
Resorcinol/formaldehyde molar ratios	0.6/2.2		
Solidification temperature, °C	210 °C		
Surface morphology	non-porous	porous	
Pore-forming agent	-	CaCO_3_	toluene
Pore-forming agent content, wt.%	0	10	25

**Table 6:** Compositions of simulate solutions used in the present work.

Composition (g/L)	Model Solution				
	WWER *	RBMK *	Solution No. 1	Solution No. 2	Solution No. 3
Na^+^	61.7	80.5	2.3	69.0	2.3
K^+^	15.4	16.0	-	-	-
NO_3_^−^	69.4	242.4	6.2	139.5	6.2
B (recalculated on H_3_BO_3_)	98	-	-	-	-
Total mineralization	214	339	2.3	209	2.3
pH	11–12.5	10–12	11	≥13	9

* Solution simulating the evaporator concentrate from WWER or RBMK reactor [5,6].

**Table 7:** Calculated chemical composition of the alkaline model solution simulating the clarified phase in storage tanks with heterogeneous waste of high-level activity, pH ≈ 12 [1], referred as “CP-HLW”.

Composition		Concentration in Solution (g/L)	
K^+^		0.6	
Al^3+^		6.0	
Cr^3+^		0.4	
Si^2+^		0.2	
NaOH		100	
Na^+^	from NaOH	57.5	101.2
	from salts ^1^	43.7	
NO_3_^−^	from NaNO_3_	68.7	110.0
	from salts ^2^	41.3	
NO_2_^−^		35.0	
SO_4_^2−^		1.5	
CrO_4_^2−^		0.9	
Cs^+^		0.05	

^1^ from Na_2_SiO_3,_ NaNO_3_, NaNO_2_ and Na_2_SO_4_. ^2^ from Al(NO_3_)_3_·9H_2_O.

## Data Availability

Not applicable.

## Sample Availability

Samples of the compounds are not available from authors.
